# Molecular Recognition between Aβ-Specific Single-Domain Antibody and Aβ Misfolded Aggregates

**DOI:** 10.3390/antib7030025

**Published:** 2018-07-13

**Authors:** Mingzhen Zhang, Jie Zheng, Ruth Nussinov, Buyong Ma

**Affiliations:** 1 Department of Chemical & Biomolecular Engineering, the University of Akron, Akron, OH 44325, USA; mingzhen.zhang@nih.gov; 2 Leidos Biomedical Research, Inc., Frederick National Laboratory for Cancer Research, Frederick, MD 21702, USA; nussinor@mail.nih.gov; 3 Sackler Institute of Molecular Medicine, Department of Human Genetics and Molecular Medicine, Sackler School of Medicine, Tel Aviv University, Tel Aviv 69978, Israel

**Keywords:** single-domain antibodies, Aβ peptide, amyloid antibody, Alzheimer’s disease, antibody recognition

## Abstract

Aβ is the toxic amyloid polypeptide responsible for Alzheimer’s disease (AD). Prevention and elimination of the Aβ misfolded aggregates are the promising therapeutic strategies for the AD treatments. Gammabody, the Aβ-Specific Single-domain (VH) antibody, recognizes Aβ aggregates with high affinity and specificity and reduces their toxicities. Employing the molecular dynamics simulations, we studied diverse gammabody-Aβ recognition complexes to get insights into their structural and dynamic properties and gammabody-Aβ recognitions. Among many heterogeneous binding modes, we focused on two gammabody-Aβ recognition scenarios: recognition through Aβ β-sheet backbone and on sidechain surface. We found that the gammabody primarily uses the complementarity-determining region 3 (CDR3) loop with the grafted Aβ sequence to interact with the Aβ fibril, while CDR1/CDR2 loops have very little contact. The gammabody-Aβ complexes with backbone binding mode are more stable, explaining the gammabody’s specificity towards the C-terminal Aβ sequence.

## 1. Introduction

While its normal biological function has not been fully understood, amyloid β (Aβ) peptide is involved in Alzheimer’s disease (AD) [1,2], resulting in the fatal cognitive decline, speech loss, behavioral disorders, and mood swings [3,4,5]. The aggregation of Aβ peptides follows three-phase kinetics, leading to the toxic Aβ aggregates enriched with β-sheet structures [6,7,8,9,10,11,12,13]. Previous evidence suggested that the Aβ amyloid’s neurotoxicities are due to its interference with cellular ionic homeostasis, signaling pathways, the oxidative levels via the membrane-involving mechanisms [14,15,16]. Recently, more evidence points to the neuroinflammation mechanisms mediated by microglial cells. It has been found that the microglia cell receptor TREM2 binds quite specifically to Aβ peptide, particularly to Aβ peptide oligomers [17,18]. The TREM2 mutations have also been shown to significantly increase Alzheimer’s risk, indicating their fundamental role in protecting the brain [19]. 

Tremendous efforts have been made in developing Aβ inhibitors as AD therapies in the past decades [20]. Various Aβ inhibitors have been investigated, including the small organic chemical compounds (epigallocatechin gallate (EGCG) [21], tanshinones [22], curcumin [23]), nanoparticles (Nano-N2PY [24], NIPAM [25], AuNPS [26]), peptides (IAPP segments [27,28,29,30,31], Aβ segments [32], non-natural hexapeptides [33,34,35]), and the peptides mimetics (*N*-methylated IAPP sequences [36], macrocyles [37]). The immunotherapeutic approaches attracted increasing interest for AD treatment [38,39,40,41,42,43,44]. Pre-clinical neuropathological examinations suggested that the engineered antibodies, including solanezumab [45], bapineuzumab [46], gantenerumab [47], ponezumab [48], gammagard [49], and octagam [50], exhibit the high efficiencies in cleaning Aβ plaques and improving the performance of the AD symptoms in the animal and in vitro models. However, these therapeutic antibodies fail to arrest the cognitive declines of AD patients in late-stage clinical trials [51,52,53]. Besides the above large antibody, smaller single domain nanobody also has been tested to prevent Aβ amyloid formation. Tessier and co-workers designed series of conformational-specific amyloid-motif antibodies (gammabodies) by grafting small Aβ amyloidogenic motifs into the complementarity-determining regions (CDRs) of a single-domain antibody (VH) [54,55,56]. The engineered gammabodies with nanometer molecular size present excellent antigen binding affinity [55]. The grafted amyloid sequences in CDR region recognize Aβ monomers, oligomers and fibers, and reduced Aβ toxicities [55]. The grafting of highly hydrophobic and amylogenic motifs increases self-aggregations propensity of gammabody [56,57,58]. Introducing charged residues may improve the solubility and eliminate the self-aggregation of the Aβ-specific gammabodies [59]. Introducing arginine in the gammabody CDR displays context-dependent affinity/specificity trade-offs [60], consistent with the trend revealed by our structural analysis of antibody-antigen interactions [61]. Overall, it is challenging to eliminate the self-aggregation tendency of the Aβ sequences in CDRs and meanwhile maintain its ability to recognize the Aβ aggregates, which requires a comprehensive understanding of the gammabody-Aβ recognition.

While there are many crystal structures of antibodies binding with monomeric amyloidogenic peptides [62], there is no structural information of antibody in complex with aggregated protein. We recently identified the binding modes of stable complexes of crenezumab with Aβ pentamer (oligomer model) and 16-mer (fibril model), providing molecular insight into the antibody-amyloid recognition mechanism [63]. Here, we examine the nanobody-amyloid interaction to see the similarity with and difference from the larger antibody-amyloid recognition. We employed the explicit-solvent all-atom molecular dynamics simulations to study the molecular recognitions between gammabodies comprising the C-terminal Aβ segment (residues 33–42) and Aβ amyloidogenic aggregates. Among many heterogeneous binding modes, our results suggest that the gammabody may recognize the Aβ aggregates via both backbone and side-chain surface interaction, with the backbone β-sheet interaction preferred. Seven grafted Aβ residues show the dominant energy contributions to the gammabody-Aβ recognitions, in line with the experimental results [55].

## 2. Materials and Methods

### 2.1. Gammabody Grafted with the C-Terminal 33–42 Aβ Residues

**Figure 1:**
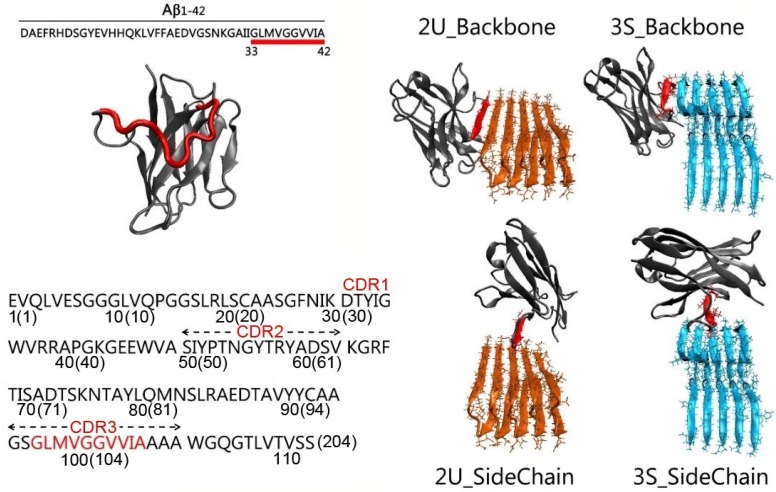
Structures and sequences of the gammabody, Aβ and their complexes investigated. Color Code: grafted Aβ residues in gammabody (red), the gammabody scaffold (gray), 2U Aβ aggregates (orange), 3S Aβ aggregates (cyan). The complementarity-determining regions (CDRs) are defined using Kabat numbering, the consecutive numbers are indicated in parenthesis. Two Aβ conformations are considered here: 2U Aβ model comprising two parallel β-sheet strands connected by one turn region, forming a U-shape conformation, and the 3S Aβ model with three parallel β-sheet strands bridged by two turn regions.

Initial coordinates of the gammabody were modeled based on the crystal structure of the VH domain of an antibody (PDB:3B9V) [58]. The residues WGGDGFYAMDY in the CDR3 regions of the native antibody VH domain were replaced by the C-terminal 33–42 residues (GLMVGGVVIA, Figure 1) [59]. The modeled gammabody was optimized by (i) with the backbone of the gammabody scaffold constrained, the grafted Aβ sequences in CDR3 were minimized by a 2000 step minimization and a short 3 ns gas phase simulation with the gammabody scaffold backbone constrained; (ii) a short 3 ns simulation in the water box, with the protein backbone constrained; (iii) a 3 ns simulation without any constraints, under a time-step of 1 fs. Finally, the modeled gammabody was subject to the explicit-solvent all-atom simulations.

### 2.2. Misfolded Aβ Aggregates

Aβ misfolded aggregates have polymorphologies [6,64]. Two typical Aβ conformations were employed here, i.e., the U-bent double β-sheet (2U) [65] and shape three β-sheet (3S) [66]. The initial coordinates of two Aβ pentamers were obtained from protein data bank (PDBID of 2BEG for 2U [65] and 2MXU for 3S [67]). The 2U and 3S Aβ models had the different structural arrangements [65,68]. The 2U Aβ model comprised two parallel β-sheet strands connected by one turn region, forming a U-shape conformation [65]. The 3S Aβ model exhibited three β-sheet strands bridged by two turn regions. 

### 2.3. Gammabody-Aβ Complexes

The gammabody recognizes the Aβ aggregates in the homotypic manner [55]. The initial binding interfaces in gammabody-Aβ complexes were constructed by the in-register residue interactions between the Aβ aggregates and the grafted Aβ sequences in gammabody. For a given Aβ aggregates recognized by the gammabody, two modes were considered, i.e., the backbone scenario in which the grafted Aβ sequences in gammabody binding to the identical residues in the Aβ aggregates via the backbone contacts, and the side-chain scenario in which the Aβ motif in gammabody interacted with the Aβ aggregates via the side-chain contacts [69]. For clarity and convenience, the modeled gammabody-Aβ complexes were denoted by the type of the Aβ aggregates followed by the gammabody-Aβ recognition model, i.e., 2U_Backbone, 2U_SideChain, 3S_Backbone, and 3S_SideCahin, respectively (Figure 1).

In both 2U_Backbone and 3S_Backbone complexes, the gammabody-Aβ recognition interfaces were constructed by the in-register residue interactions between ^33^G_gammabody_–^33^G_Aβ_, ^34^L_gammabody_–^34^L_Aβ_, ^35^M_gammabody_–^35^M_Aβ_, ^36^V_gammabody_–^36^V_Aβ_, ^37^G_gammabody_–^37^G_Aβ_, ^38^G_gammabody_–^38^G_Aβ_, ^39^V_gammabody_–^39^V_Aβ_, ^40^V_gammabody_–^40^V_Aβ_, ^41^I_gammabody_–^41^I_Aβ_, and ^42^A_gammabody_–^42^A_Aβ_ via the backbone contacts (For easy comparison, the residue numbers in grafted Aβ motif in gammabody are mentioned as their original residue number in Aβ peptide). The initial distance between the Aβ motif in gammabody and the Aβ aggregate was set to 4.75 Å, similar to the peptide-peptide distance in the Aβ fibril structures. For 2U_SideChain complex, the initial recognition interfaces were constructed by the sidechain contacts between ^33^G_gammabody_–^41^I_Aβ_, ^34^L_gammabody_–^40^V_Aβ_, ^35^M_gammabody_–^39^V_Aβ_, ^36^V_gammabody_–^38^G_Aβ_, ^37^G_gammabody_–^37^G_Aβ_, ^38^G_gammabody_–^36^V_Aβ_, ^39^V_gammabody_–^35^M_Aβ_, ^40^V_gammabody_–^34^L_Aβ_, and ^41^I_gammabody_–^33^G_Aβ_. This hydrophobic interface has been proved to be stable peptide-peptide interfaces in the Aβ double-layer aggregates [67]. The 3S_SideChain complex had the curved surface. Tcl scripts were used to model a curved conformation for the grafted Aβ motif in gammabody to match the non-flat surfaces in 3S Aβ aggregates, with the initial interfacial interactions of ^34^L_gammabody_–^42^A_Aβ_, ^35^M_gammabody_–^40^I_Aβ_, ^36^V_gammabody_–^39^V_Aβ_, ^37^G_gammabody_–^38^V_Aβ_, ^38^G_gammabody_–^37^G_Aβ_, ^39^V_gammabody_–^36^G_Aβ_, ^40^V_gammabody_–^35^V_Aβ_, and ^41^I_gammabody_–^34^M_Aβ_, and ^42^A_gammabody_–^33^L_Aβ_. To optimize the modeled gammabody-Aβ complexes, a 5000 steps minimization was conducted with the backbone atoms of the Aβ aggregations and the grafted Aβ motif in gammabody constrained, followed by a short 2 ns simulations with the time-step of 1fs in gas phase. Then, all the optimized gammabody-Aβ complexes were solvated in the explicit-solvent box with the ion concentration of ~0.15 M. The minimization of 5000 steps was conducted. The short 3 ns explicit-solvent simulations with the protein backbone constrained, and the 3 ns simulations without any constraints were conducted to fully relax the water and ion atoms.

### 2.4. All-Atom MD Simulation Protocol

The simulations were conducted in the GPU-accelerated Gromacs-4.5.3 package with the explicit TIP3 solvent model using the all-atom CHARMM27 force filed and CMAP correction [70]. Counter ions of NaCl were added to the systems to neutralize and mimic ~0.15 M ionic strength. The short constant-temperature, constant-pressure (NPT) simulations were performed to gradually head the systems from 0 to 310 K and approach the pressure of 1 atm under periodic boundary condition. The hydrogen-involved covalent bonds were constrained by the LINCS method. Short-range Van der Waals (VDW) interactions were calculated by a smoothly truncated function with a potential shift at 14 Å, while long-range electrostatic interactions were calculated by the particle mesh Ewald (PME) method with a grid space of 0.16 Å and a real space cutoff of 12 Å. The velocity Verlet integration was used to generate a 2 fs time-step in all the simulations. All analyses were performed using the tools within the CHARMM, VMD, and in-house codes.

## 3. Results and Discussion

### 3.1. Structure and Dynamics of the Grafted Gammabodies

The CDR sequences in antibody defined the binding specificity [55]. While Aβ sequences were introduced into the CDR3 region, the gammabodies show the capacities of recognizing the Aβ aggregates. The gammabodies with the N-terminal sequences of Aβ peptide cannot recognize Aβ aggregates, and those with the central hydrophobic Aβ segments weakly bind to the Aβ mature fibrils. In contrast, the gammabodies with the C-terminal segments of Aβ strongly recognize the Aβ monomer, oligomers and fibers, eliminating the misfolded Aβ deposits and reducing their toxicities [55]. The highest immunological ability was achieved by the gammabody with the C-terminal Aβ residues 33–42 (GLMVGGVVIA), which binds the Aβ oligomers and fibers with affinity of 5.8 and 2.4 nM, respectively [55].

**Figure 2:**
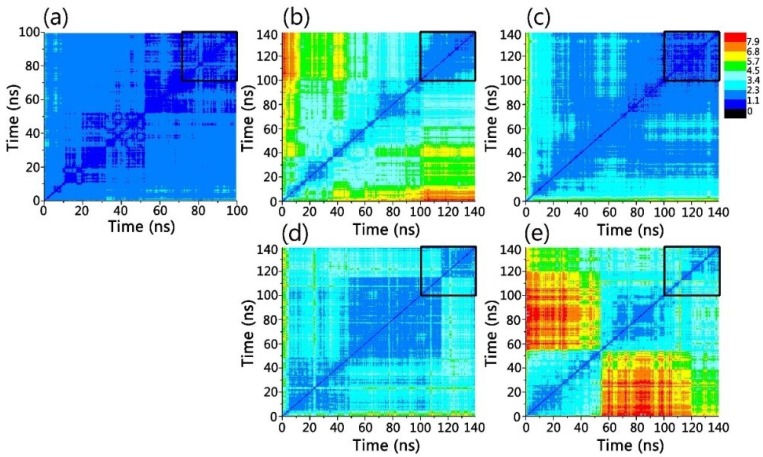
Two-dimensional root-mean-square deviation (2D-RMSD) matrix indicated that simulated system reached conformation equilibrium in 100–140 ns simulations. (**a**) gammabody; (**b**) 2U_Backbone; (**c**) 2U_SideChain; (**d**) 3S_Backbone; and (**e**) 3S_SideChain gammabody-Aβ complexes. The black boxes at the top right corner highlight the RMSD values in the equilibrium trajectories.

**Figure 3:**
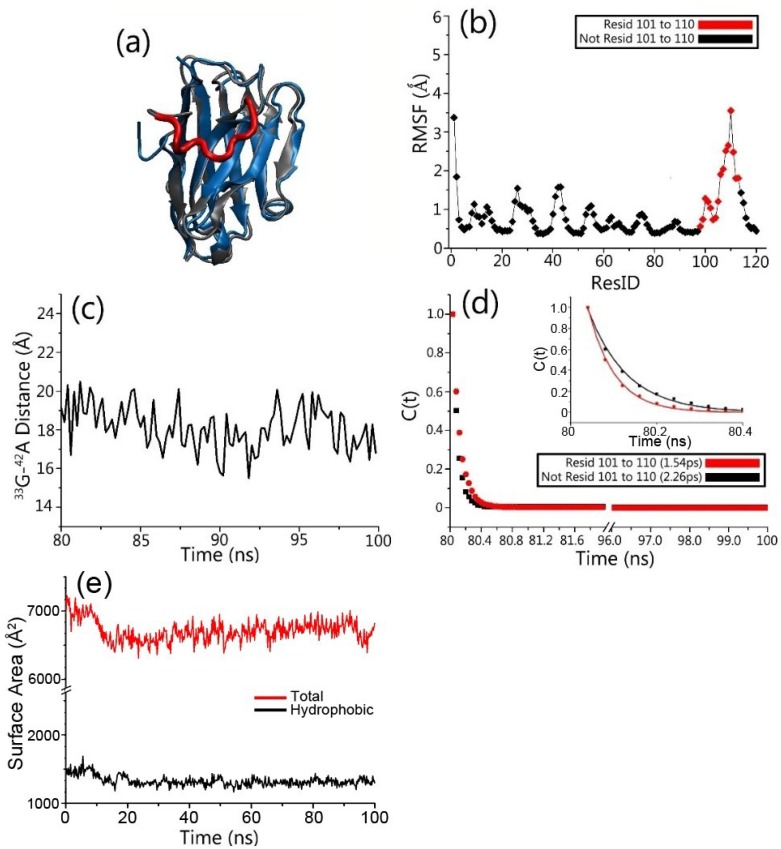
The CDR3 loop is highly flexible while gammabody framework is stable during simulations of gammabody in apo form. (**a**) Superimposed modeled structures of the native (blue ribbon) and engineered gammabody (gray and red ribbon); (**b**) residue-based RMSF profile; (**c**) ^33^G–^42^A distance for grafted Aβ sequences in gammabody; (**d**) the solvent water molecule residence time profiles for engineered gammabody with Aβ residues; and (**e**) Total solvent accessible surface area (SASA) of gammbody for non CDR3 regions, red line: total SASA, black line: hydrophobic SASA. RMSF: root-mean-square fluctuation.

Examining the structural and dynamic properties of the engineered gammabody in apo form shows that grafted Aβ motif did not disturb the overall structure of the gammabody. The two-dimensional root-mean-square deviation (2D-RMSD) matrix (Figure 2a) indicates that simulated gammabody experienced the minor structural change and achieved equilibrium quickly, with the overall secondary structures and the conformation preserved. Its RMSD values, relative to its native (PDB:3B9V [58]) conformation, fluctuate around 2.35 Å (Figure 2a and Figure 3a). We also monitored the change of solvent accessible area for gammabody, not including CDR3 region. As can be seen in Figure 3e, the exposed hydrophobic surface area maintains a stable value during the simulation. Comparing to its parent scaffold, the grafting of Aβ motifs increases gammabody’s self-aggregations propensity [56,57,58]. The structural stability of the framework residues and constant exposed hydrophobic surface area observed in the simulation suggests that the self-aggregations of the gammabody may not be triggered by destabilization of the framework. However, our current study does not address self-aggregation of gammabody, which could be an interesting topic in future computational study.

The root-mean-square fluctuation (RMSF) values for gamabody scaffold are ~1 Å (Figure 3b). The grafted Aβ motif in gammabody with the extended coil conformation are more flexible, with the higher RMSF values up to 3.5 Å. The end-end distances are ~18 Å (Figure 3c), due to the restriction of framework residues. The extended conformation of the grafted Aβ motif may help its binding with β-strands of Aβ aggregates.

We calculated the water residence times (*τ_s_*) around the gammabody to evaluate the hydration shells.

(1) $$C_{R}\left(t\right)=\frac{1}{N_{w}}\sum_{j=1}^{N_{water}}\frac{P_{j}\left(0\right)P_{j}\left(t\right)}{P_{j}{\left(0\right)}^{2}}$$

By fitting the correlation functions Equation (1) using the exponential function:

(2) $$C_{R}\left(t\right)=B+A\exp\left(-t/\tau_{s}\right)$$

where *N_w_* is the number of water molecules within 10 Å of the targeting segments and *P*(*t*) is the binary function that determines the existence of the water molecules within 10 Å of the targeting segments, the residence time (*τ_s_*) was calculated. The residence times reflects the stability of the hydration shells around the protein surfaces. A larger *τ_s_* represents a stronger hydration shell (smaller solvation entropy), and vice versa. In line with the higher structural flexibility, the grafted Aβ sequences in gammabody presented the weaker hydration shell (residence time: 1.54 ps) compared to that of gammabody scaffold (residence time: 2.26 ps) (Figure 3d). The weak hydration shell of the grafted Aβ motif suggested that it may incur less desolvation energy upon binding to Aβ aggregates.

### 3.2. Gammabody-Aβ Recognitions

Aβ aggregates have polymorphologies, as evidenced by extensive experiments and simulations [6,68,71,72]. Two typical Aβ conformations with the similar β-sheet structure, but the different arrangements were employed, i.e., the U-bent double β-sheet (2U) and S-shaped triple β-sheet (3S) [66] (Figure 1). Mimicking the Aβ self-aggregation process [73], two recognition scenarios, backbone and sidechain contacts, were used to generate a total of four gammabody-Aβ complexes (Figure 1). We performed 140 ns MD simulations to test their structural stabilities.

2D-RMSD plots in Figure 2 show that the simulated gammabody-Aβ complexes experienced the structural relaxations and achieved the equilibrium after 100 ns. The 2D-RMSD plots in equilibrium trajectory indicate that the simulated 2U_Backbone and 2U_SideChain complexes showed the higher overall stability with the averaged RMSD of ~1.5 Å, compared to the 3S_Backbone and 3S_SideChain complexes with the RMSD around ~2.6 Å, respectively.

**Figure 4:**
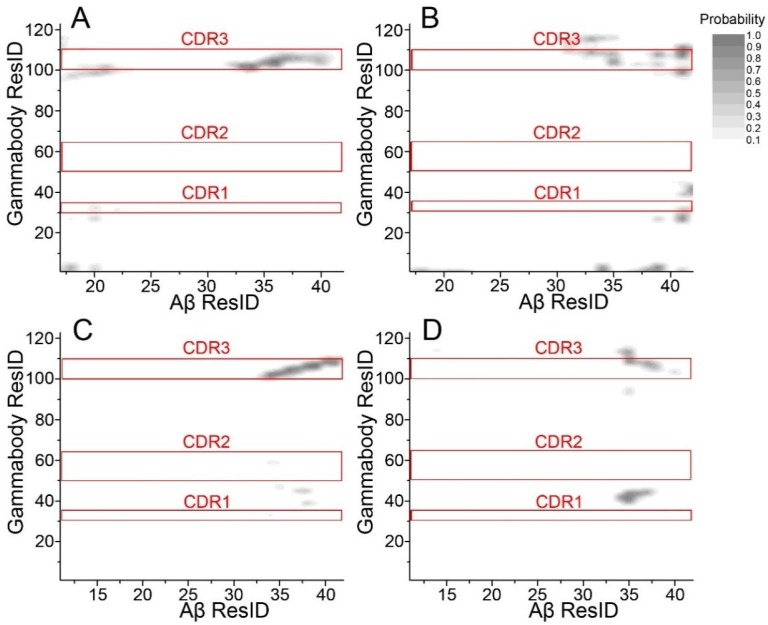
The gammabody mainly uses the CDR3 to interact with Aβ amyloid, while there is no contributions from the CDR1/CDR2 residues. Residue contact maps for (**A**) 2U_Backbone; (**B**) 2U_SideChain; (**C**) 3S_Backbone; and (**D**) 3S_SideChain complexes. Two residues at the gammabody-Aβ interface with non-hydrogen atom distance <5 Å are defined as the contacting residues.

An important question to be answered is that if CDR1 and CDR2 residues also contribute to the gammabody-Aβ recognition. We examined atomic contact between non-hydrogen atoms of gammabody and Aβ aggregates. As can be seen in Figure 4, there is no specific interactions between CDR1/CDR2 residues and Aβ aggregates. Grafted Aβ motif in CDR3 forms stable interaction with β-sheet backbone of Aβ aggregates. However, when binding to the sidechain surface, the CDR3 residues and other framework residues interact non-specifically with C-terminal residues of Aβ aggregates.

**Figure 5:**
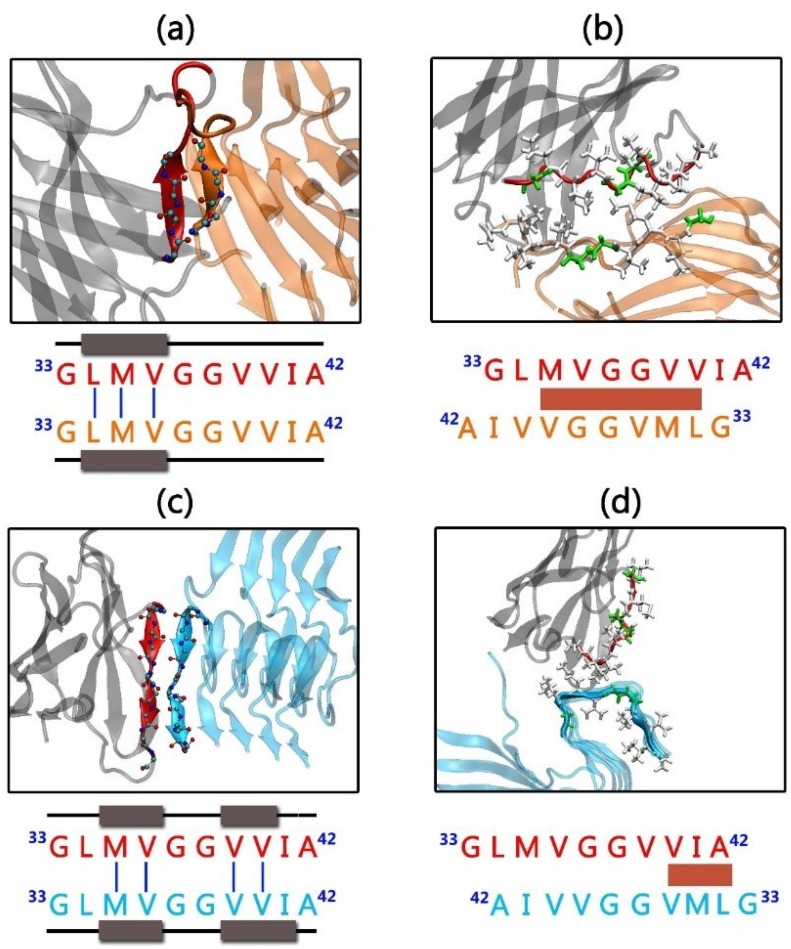
Simulation snapshots suggest that the gammabody CDR3 loop can form stable β-sheet interaction with Aβ amyloid, and binding on sidechain surface is also possible. Interfacial interactions of (**a**) 2U_Backbone; (**b**) 2U_SideChain; (**c**) 3S_Backbone; and (**d**) 3S_SideChain gammabody-Aβ complexes, with the interfacial interactions highlighted. For easy comparison, the residue numbers in grafted Aβ motif in gammabody are set as their original residue number in Aβ peptide. Gammabody residues are in red color.

**Figure 6:**
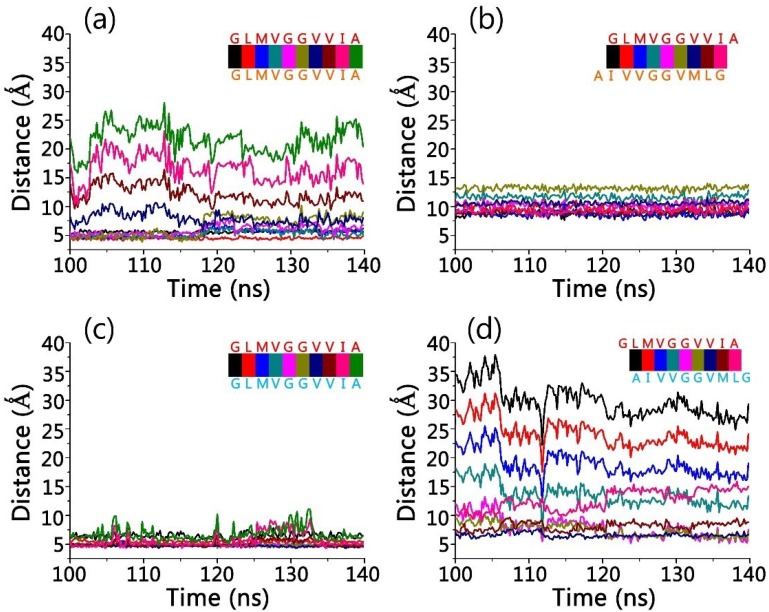
Several key residues contribute to the stable gammabody-Aβ amyloid interactions. Interfacial residue-residue distance profiles for (**a**) 2U_Backbone; (**b**) 2U_SideChain; (**c**) 3S_Backbone; and (**d**) 3S_SideChain gammabody-Aβ complexes.

**Figure 7:**
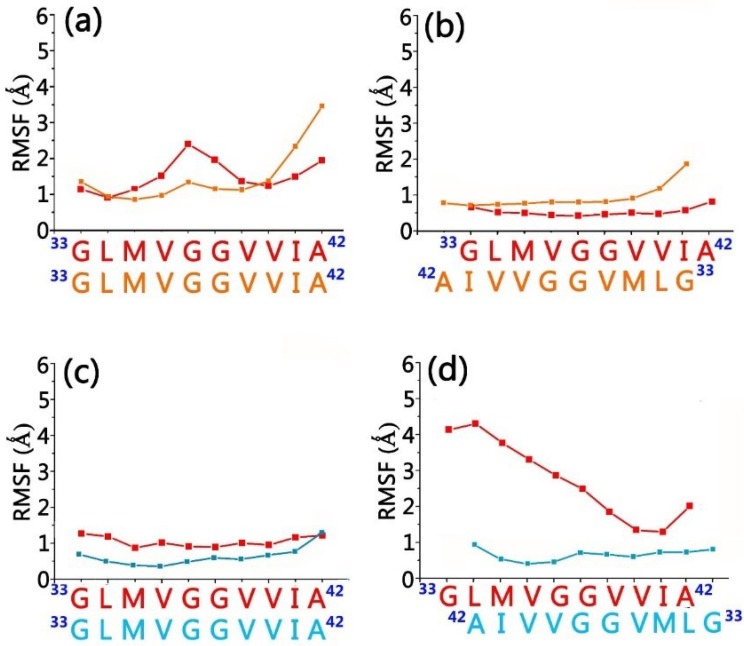
Residue-based RMSF profiles indicated that CDR3 loop cannot be stabilized when binding on the sidechain surface of 3S Aβ amyloid structure. (**a**) 2U_Backbone; (**b**) 2U_SideChain; (**c**) 3S_Backbone; and (**d**) 3S_SideChain gammabody-Aβ complexes. For easy comparison, the residue numbers in grafted Aβ motif in gammabody are set as their original residue number in Aβ peptide. Color Codes: the grafted Aβ residues in gammabody (red), 2U Aβ aggregates (orange), and 3S Aβ aggregates (cyan).

Upon recognitions, gammabodies and Aβ aggregates have minor overall conformational change. Figure 5 shows the final structures for all the simulated systems, in which all the Aβ misfolded aggregates preserved the amyloidogenic in-register β-sheet conformations. As expected, The RMSD values for grafted Aβ motif in gammabody is higher (2.8–4.1 Å) than gammabody scaffold (2.2–2.3 Å). The residue-residue distance profiles were calculated to evaluate the residue interactions at the gammabody-Aβ interfaces (Figure 6). For the 2U_Backbone complex, the in-register ^33^G_gammabody_–^33^G_Aβ_, ^34^L_gammabody_–^34^L_Aβ_, ^35^M_gammabody_–^35^M_Aβ_, and ^36^V_gammabody_–^36^V_Aβ_ interactions showed the distances fluctuate around 5 Å, while others were much larger. The non-bond interaction and secondary structure analysis indicate that these residues may form β-sheets at the gammabody-Aβ interface (Figure 5a). The RMSF values for these residues are 0.9–1.6 Å, verifying their good stabilities (Figure 7a). For 2U_Sidechain complex, a stable interface was identified. The interfacial residue pairs displayed the steadier and lower residue distance profiles (Figure 6b). The gammabody-Aβ interface was established by the strong hydrophobic contacts. The RMSF values for the interfacial residues are consistently lower than 2.0 Å (Figure 7b).

The 3S_Backbone complex showed the strong interaction between gammabody and Aβ aggregates, with the low residue distance profiles of ~5.0–7.0 Å, and their residue-based RMSF values are consistently smaller than 1.5 Å (Figure 7c). This suggests a perfect parallel residue alignment at the recognition interfaces. The snapshot of the recognition interfaces in 3S_Backbone model, combined with the secondary structure and the hydrogen bonding analysis, showed that the intermolecular backbone hydrogen bonds at two regions of 34L–36G and 39V–41I facilitated the formation of two β-sheet segments at the interfaces that occupied ~60% total areas of the recognition segments (Figure 5c). Due to the non-flat surface of 3S Aβ aggregates, the binding between the gammabody and 3S Aβ aggregate via the side-chain contacts was less stable, with the small contacting area. The hydrophobic contacts between 41I–42A of gammabody and 34L–37V of Aβ aggregates maintained the gammabody-Aβ interface (Figure 5d and Figure 6d). The RMSF values of interfacial residues are 0.6–1.4 Å (Figure 7d). 

### 3.3. Interaction Energies between Gammabody and Aβ Aggregates

To evaluate the contributions of the interfacial residues to the gammabody-Aβ recognitions, the residue-based interaction energies for the simulated gammabody-Aβ complexes were calculated. For 2U_Backbone complex, the energy contribution of C-terminal Aβ sequences in gammabody (33G–38G) ranged from −6.8 to −18.9 kcal/mol (Figure 7). Similarly, the C-terminal Aβ sequences in gammabody (33G–40V) also have the stronger contributions to the interface of 3S_Backbone complex. These residues appear to have the comparable interaction energies ranging from −6.0 to −14.3 kcal/mol. In both 2U_SideChain and 3S_SideChain complexes, three residues at the C-terminal Aβ sequences in the gammabody (40V, 41I and 42A) exhibited the higher interaction energies (−4.4 to −12.5 kcal/mol) than others.

**Figure 8:**
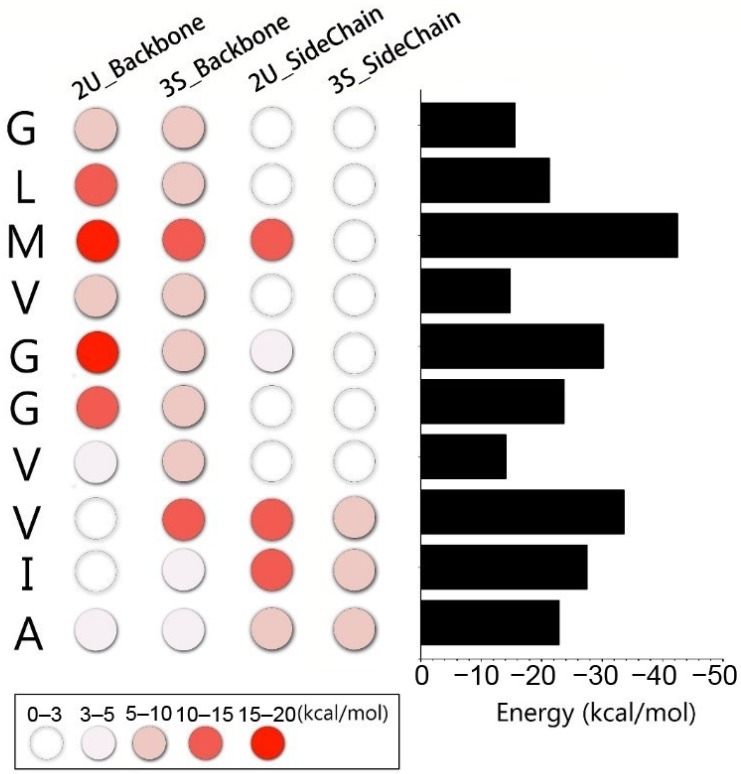
Residue-based interaction energies between gammabody with Aβ aggregates suggest that M35 could be the most important residue in the grafted Aβ sequence to interact with Aβ amyloid. The residue color scheme was based the absolute value of interaction energy. The summed residue interaction energies for four gammabody-Aβ complexes are shown in the right panel. The interaction energies were based on the average structure from the last 40 ns simulations.

Since the recognition between gammabody and Aβ oligomers in the solutions may have different binding modes, the residual interaction energies of four simulated systems were summed up to identify the important residues towards the gammabody-Aβ recognitions. Although the computational residue interaction energies may not exactly reflect the populations, these results estimate the general energetic preference for the interfacial residues. As shown in Figure 8, seven residues displayed the predominant interaction energies ranging from −21.2 to −42.4 kcal/mol. These results are consistent with the experimental implications [55]. Previous assays have shown that while gammabodies containing Aβ34–39 retain the ability to recognize various Aβ aggregates, removing of 34 and 35 from residue 34–39 region makes gammabody completely inactive for the Aβ recognitions, emphasizing the significance of the 34L and 35M in gammabody-Aβ binding [55]. Meanwhile, while three residues at C-termini (40V, 41I and 42A) were added into the inactive gammabody with Aβ 36–39 regions, the gammabody with Aβ 37–42 residues recovers its strong ability to bind the Aβ aggregates, indicating that the 37G, 40V, 41I and 42A are indispensable for the Aβ aggregations [55]. Further comparison between the immunological active gammabody with Aβ 37–42 and inactive gammabody with 39–42 also suggested that the presence of residue 37G and 38G are crucial for the gammabody-Aβ recognitions [55]. Thus, both the computational and experimental results suggested the 34L and 35M, 37G, 38G, 40V, 41I and 42A could be the key residues for the gammabody-Aβ recognitions.

To gain thermodynamic insights into the recognition scenarios, the MM-GBSW algorithms were used to calculate the binding energies between gammabody and Aβ aggregates. As shown in Table 1, gammabody has a stronger binding affinity with the 2U Aβ fibril structure (−64.2 to −64.8 kcal/mol) than the 3S structure (−25.7 to −30.0 kcal/mol). It is interesting to note that the complexes with backbone binding mode have the lower total energy than non-specific binding on the Aβ aggregates sidechain surface, for both 2U and 3S structures, respectively. The trend is consistent with the residue interaction energy analysis in the Figure 8, indicating that the gammybody has better interaction with Aβ aggregates when stable β-sheet structure is available.

Amyloid oligomer can be the subunit of amyloid fiber [74], and theAβ oligomers are equally rich in the solvent-exposed backbones and side chain atoms [74,75]. Although the recognition of either Aβ oligomers or fibers by the gammabodies could be the combination of both elongation and lateral manners, the stronger association through backbone extension of stable β-sheet implies specificity towards the C-terminal Aβ sequence.

## 4. Conclusions

Aβ is the causative agent for AD and is present in over 80% of AD patients. Prevention of Aβ misfolding and the clearance of the accumulated Aβ aggregates are promising strategies for AD treatment. The Aβ-specific antibodies attract attention due to their high immunological efficiency and specificity to the Aβ misfolded aggregates. In this work, we investigated various gammabody-Aβ complexes with four recognition scenarios to answer two questions: (1) which is the preferred binding mode for the gammabody-amyloid interaction, extension of beta-sheet interaction or binding on the sidechain surface? (2) Do CDR1/CDR2 loops also contribute to gammabody-amyloid interaction? Our results suggest that the gammabody can bind Aβ misfolded aggregates via the backbone and side-chain contacts. However, the gammabody-Aβ complexes with backbone binding mode may be more stable, explaining the gammabody’s specificity towards the C-terminal Aβ sequence. We propose that the gammabody primarily uses the CDR3 loop with the grafted Aβ sequence to interact with the Aβ fibril, while CDR1/CDR2 loops have very little contact. This binding mode is understandable, but in terms of shape is different to common antibody-antigen bindings, which usually employ multiple antibody loops to interact with antigen. Future engineering to improve CDR1/CDR2 contribution may be helpful.

## Figures and Tables

**Table 1:** Binding energies for 2U_Backbone, 2U_SideChain, 3S_Backbone, and 3S_SideChain gammabody-Aβ complexes.

System	ΔG_gas_ (kcal/mol)	ΔG_sol_ (kcal/mol)	TΔS (kcal/mol)	E_total_ (kcal/mol) ^a^	ΔG_binding_^b^ (kcal/mol)
2U_Backbone	−363.7 ± 32.1	273.6 ± 30.7	−26.0	−1442.9 ± 49.0	−64.2 ± 8.6
2U_SideChain	−283.9 ± 16.2	193.1 ± 15.1	−25.9	−1431.9 ± 45.4	−64.8 ± 5.5
3S_Backbone	−117.6 ± 14.7	61.5 ± 13.8	−26.1	−1974.1 ± 48.0	−30.0 ± 4.9
3S_SideChain	−94.3 ± 10.1	42.5 ± 6.4	−26.1	−1968.4 ± 50.2	−25.7 ± 6.3

^a^ Aβ17–42 is used in the 2U structure, and Aβ11–42 is used in the 3S structure; ^b^ Binding energy: <ΔG_binding_> = <ΔG_gas_> + <ΔG_sol_> − <TΔS>. 200 conformations from last 40 ns simulations were used in the energy evaluation.
